# An Updated Perspective on Current Prognostic and Predictive Biomarkers in Chronic Lymphocytic Leukemia in the Context of Chemoimmunotherapy and Novel Targeted Therapy

**DOI:** 10.3390/cancers12040894

**Published:** 2020-04-07

**Authors:** Jared A. Cohen, Riccardo Bomben, Federico Pozzo, Erika Tissino, Andrea Härzschel, Tanja Nicole Hartmann, Antonella Zucchetto, Valter Gattei

**Affiliations:** 1 Clinical and Experimental Onco-Hematology Unit, Centro di Riferimento Oncologico di Aviano (CRO) IRCCS, via Franco Gallini 2, 33081 Aviano, Italyrbomben@cro.it (R.B.); fpozzo@cro.it (F.P.); etissino@cro.it (E.T.); vgattei@cro.it (V.G.); 2 Department of Internal Medicine I, Medical Center and Faculty of Medicine, University of Freiburg, Hugstetter Str. 55, 79106 Freiburg, Germany; andrea.haerzschel@uniklinik-freiburg.de (A.H.); tanja.hartmann@uniklinik-freiburg.de (T.N.H.)

**Keywords:** CLL, prognosticator, predictor, CD49d, VLA-4

## Abstract

Chronic lymphocytic leukemia (CLL) is a heterogeneous disease with a variable clinical course. Novel biomarkers discovered over the past 20 years have revolutionized the way clinicians approach prognostication and treatment especially in the chemotherapy-free era. Herein, we review the best established prognostic and predictive biomarkers in the setting of chemoimmunotherapy (CIT) and novel targeted therapy. We propose that TP53 disruption (defined as either *TP53* mutation or chromosome 17p deletion), unmutated immunoglobulin heavy chain variable region gene status (UM IGHV), *NOTCH1* mutation, and CD49d expression are the strongest prognosticators of disease progression and overall survival in the field of novel biomarkers including recurrent gene mutations. We also highlight the predictive role of TP53 disruption, UM IGHV, and *NOTCH1* mutation in the setting of CIT and TP53 disruption and CD49d expression in the setting of novel targeted therapy employing B-cell receptor (BCR) and B-cell lymphoma-2 (BCL2) inhibition. Finally, we discuss future directions in the field of biomarker development to identify those with relapsed/refractory disease at risk for progression despite treatment with novel therapies.

## 1. Introduction

Chronic lymphocytic leukemia (CLL) is a heterogeneous disease with a variable clinical course. Over the last 20 years, the discovery of novel biomarkers has revolutionized disease prognostication and treatment prediction; however, despite the development of risk calculators such as the CLL international prognostic index (CLL-IPI) and [1,2,3,4] recently updated guideline recommendations by the NCCN [1,2,3,4,5], the precise hierarchical value of these markers remain in question, particularly in the era of targeted therapy. 

Today’s most well-established prognostic biomarkers in CLL are outlined in Table 1. They range from host factors (i.e., gender and age) to disease markers (i.e., Rai and Binet staging), antigen expression (i.e., CD38, ZAP70, and CD49d/VLA-4), serology (i.e., lactate dehydrogenase, beta-2-microglobulin [B2M], and thymidine kinase), genetics (i.e., deletion of the short arm of chromosome 17 [del17p] and *TP53* gene mutation) and immunogenetics (i.e., immunoglobulin heavy chain variable region [IGHV] gene mutational status). While some of these markers are prognostic, others are both prognostic and predictive [4,6,7,8,9,10].

Prognostic biomarkers, by definition, evaluate risk of disease progression and death and aid clinicians in aspects of patient counseling including determining frequency of follow-up and identifying those appropriate for risk-adapted early treatment. On the other hand, predictive biomarkers forecast disease response to specific treatments and are clinically useful in tailoring therapy. The purpose of this manuscript is to review well-established and novel biomarkers in CLL discussing their roles as prognostic and/or predictive biomarkers. In the course of this discussion, we aim to review current treatment options and propose a refinement of existing treatment algorithms to more accurately reflect our current knowledge.

## 2. Today’s Most Important Prognostic Biomarkers

In 2013, we performed a large meta-analysis involving 2972 cases of CLL from 8 published studies [11,12,13,14,15,16,17,18] to determine the hierarchy of 9 established prognostic biomarkers (age > 65 years, unmutated IGHV gene status, del17p, male sex, absolute lymphocyte count > 15 × 10^9^/L, ZAP70, B2-microglobulin > upper limit of normal, CD38 and del11q) with respect to overall survival (OS) in two different models considering the inclusion and exclusion of CD49d expression as a covariate [19]. Using a training/validation strategy to determine a threshold for CD49d expression at 30%, we assessed the relative prognostic value of each biomarker employing a comprehensive multivariable Cox model including stepwise elimination of nonsignificant variables. In the model excluding CD49d, UM IGHV gene mutational status, del17p, ZAP70, and CD38 were independent prognosticators of OS, however, when CD49d was included, ZAP70 and CD38 lost their independent prognostic value. A subgroup analysis of the flow-cytometry based markers (CD49d, ZAP70, CD38) using recursive partitioning and bivariate survival curves confirmed the superior performance of CD49d. This study proved that CD49d is the strongest flow cytometry marker for OS and should be considered alongside del17p and unmutated IGHV gene mutational status as the most potent biologic prognosticators.

Since the publication of that study, several recurrent gene mutations including *TP53*, *SF3B1*, *NOTCH1* and *BIRC3* have emerged as negative prognosticators including in cases of relapsed/refractory CLL [20,21,22,23,24,25,26,27]. In a 2016 follow-up study, we investigated the prognostic strength of the well-established biologic markers in the presence of these novel mutations in series of 778 CLL patients [28]. CD49d expression again prevailed in this setting, together with TP53 disruption (defined as either *TP53* gene mutation and/or del17p), UM IGHV gene mutational status and mutated *NOTCH1* with respect to OS. CD49d expression added further prognostication as a covariate in the context of the integrated hierarchical mutational/cytogenetic model proposed by Rossi et al [29] in which 4 risk categories are defined as low (del13q), intermediate (normal karyotype or tri12), high (*NOTCH1* mutation, and/or *SF3B1* mutation and/or del11q) and very high (BIRC3 disruption and/or TP53 disruption).

Taken together, these results propose that TP53 disruption, UM IGHV gene mutational status, mutated *NOTCH1* and CD49d expression are the most powerful prognosticators in CLL. 

## 3. Discussing the Role of These Prognosticators as Predictive Biomarkers

Chapters from 3.1 to 3.4 are respectively focused on discussing the role of TP53 disruption, UM IGHV mutational status, *NOTCH1* mutations and high CD49d expression as putative predictive markers in CLL. Given the emerging role of DNA methylation and complex karyotype in this setting, two additional chapters (3.5 and 3.6) have been added to discuss in details these novel aspects.

### 3.1. TP53 Disruption

The p53 tumor suppressor gene plays a crucial role regulating genomic stability and is universally implicated in tumorigenesis in both solid organ and hematologic malignancy [30,31,32]. The gene is located on chromosome 17p13.1; disruption of TP53 is therefore characterized by either chromosomal deletion or gene mutation with roughly one-third of disrupted cases presenting equally with mutation and deletion, mutation only and deletion only [33]. Small *TP53* mutated subclones discovered on ultra-deep next generation sequencing (NGS) account for 30–40% of all cases harboring TP53 defects and 6–5% of all cases of CLL [34,35]. Sub-clonal disease has the same unfavorable OS as clonal disease [34,35], likely owing in part to the bottleneck effect of chemotherapy which imparts an uncontested survival advantage to the mutated sub-population post-treatment [36,37]. These observations have raised concern regarding the sensitivity of the current agreed-upon allele frequency cut-off of 10–15% for detection of *TP53* mutated disease and selection of appropriate initial therapy in subclonal cases.

According to most practice guidelines today, TP53 disruption remains the lone predictive biomarker in CLL [38,39,40,41] and should be analyzed prior to treatment initiation in all patients owing to the large body of evidence demonstrating that patients either do not respond to initial chemoimmunotherapy (CIT) or experience relapse soon after remission [42] (Table 2).

The most comprehensive data addressing the predictive capacity of TP53 disruption comes from an analysis of the CLL-8 trial [43], a phase 3, randomized (1:1) study comparing treatment with fl a phase and cyclophosphamide (FC) or FC with rituximab (FCR) in 817 previously untreated patients in which Stilgenbauer et al., showed that patients with TP53 disruption experienced poorer clinical responses, minimal residual disease (MRD) negativity, progression-free survival (PFS) and OS after treatment with FC and FCR [44] and that anti-CD20 therapy added no OS benefit. They conclude that “17p-and *TP53^mut^* therefore define CLL patients who should be referred to specialized centers for enrollment in clinical trials developing novel treatment”. These results are in keeping with earlier findings in TP53 disrupted patients treated with chemotherapy alone using chlorambucil, fludarabine, or FC regimens [22,45,46].

The introduction of novel targeted therapy in CLL has been revolutionary, particularly benefitting ultra-high-risk patients such as those with relapsed/refractory (r/r) disease and TP53 disruption. Pathway inhibitors such as ibrutinib, a Bruton’s tyrosine kinase (BTK) inhibitor, idelalisib, a phosphoinositide 3-kinase inhibitor (PI3K), and venetoclax, a B-cell lymphoma-2 (BCL2) inhibitor, vastly outperform CIT in both response rates and PFS in cases of TP53 disrupted CLL including those with r/r disease [47,48]. For example, the overall response rates for FCR and bendamustine with rituximab (BR) in the setting of r/r disease in presence of TP53 disruption is 35% and 7%, respectively compared to 79%, 78% and 79% for ibrutinib, idelalisib with rituximab and venetoclax, respectively. Similar trends are seen for 12 and 24-month PFS as well as OS however, long-term survival data in many of these studies are still maturing [49,50,51,52,53].

Data regarding the efficacy of targeted therapy as initial treatment in patients with TP53 disruption largely come from extrapolation of data from the r/r setting however several single-arm trials [53,54,55,56] and subgroup analyses of randomized trials exist. For example, the CLL-14 trial comparing venetoclax with obinutuzumab (an anti-CD20 antibody) to chlorambucil with obintuzumab demonstrated a significantly longer 24-month PFS with the former regimen in patients with TP53 disrupted CLL [57]. Prolonged PFS was also observed in subgroup analysis of TP53 disrupted CLL in the ILLUMINATE trial favoring ibrutinib with obintuzumab to chlorambucil with obintuzumab in previously untreated patients [58].

Currently, there are no trials comparing targeted agents directly and the preferred regimens in the setting of treatment naïve (TN) and r/r TP53 disrupted CLL consist of a single small molecule inhibitor +/− anti-CD20 therapy at either indefinite or fixed-duration dosing [5].

Despite the gains made by targeted therapy in TP53 disrupted disease, data shows that even in the era of new drugs, TP53 disruption remains a negative prognosticator. In a 3 year follow up of the PCYC-1102 and -1103 trials investigating both TN and r/r CLL patients treated with single-agent ibrutinib, patients with del17p had a 30-month estimated PFS rate of 48% (95% CI, 29–65%), compared to 74% (95% CI, 53–87%) observed for del11q and 87% (95% CI, 68–95%) observed when neither aberration was present. OS rate was also shorter for del17p patients compared to del11q and patients without either cytogenetic abnormality (65%, 85% and 90%; *p* = 0.0327) [59]. These findings have also been confirmed in the setting of real-world practice treating patients with ibrutinib-based regimens [60,61]. Similar survival curves are seen for patients harboring TP53 disruption who are treated with idelalisib with rituximab [62] and venetoclax [63].

### 3.2. Immunoglobulin Mutational Status

Mutated IGHV genes are universally defined by a >2% heterogeneity in nucleotide sequence compared with germline DNA [64,65]. More recently, the use of a dichotomized cutoff of 2% heterogeneity compared to germline DNA to define M IGHV CLL has been brought into question. Jain et al., from the MD Anderson Cancer Center demonstrated a significant association with PFS and OS in 203 treatment naïve patients treated with frontline FCR when IGHV % was treated as a continuous variable with higher percentages incrementally associated with favorable PFS and OS (*p* < 0.001) [66]. A subsequent study by Morabito et al investigating the impact of IGHV % as a continuous variable on TTFT in a large cohort of 467 newly diagnosed Binet stage A patients failed to recapture the results demonstrated by the MDACC group, instead further strengthening the use of a 2% cutoff for prognostication [67].

Roughly 50% of CLL clones have an UM IGHV gene status, a feature that confers shorter OS and a higher relapse rate in the setting of FCR as is highlighted in the long-term results from non-randomized phase II trials, the randomized CLL8 trial and the subgroup analysis of the recent EGOC-ACRIN E1912 trial [44,68,69,70]. At a median follow up of 12.8 years, study authors of the original MDACC non-randomized phase II trial of FCR in 300 previously untreated patients showed a clear long-term survival benefit in those with M IGHV gene status [70]. PFS in this group was 53.9% versus 8.7% in patients with UM IGHV; of the 50.7% of patients with M IGHV who achieved MRD-negativity posttreatment, PFS was 79.8% and no relapses were observed beyond 10.4 years, arguing for the continued usage of FCR in patients with M IGHV given its potential to induce very durable remissions. This conclusion is recapitulated in the results from the mid and long-term analyses of patients from the CLL8 trial [44,68] wherein median PFS for M and UM IGHV patients was 67 and 33% respectively, at a median follow up of 5.9 years. Moreover, in both long-term studies by Thompson and Fisher the PFS curves plateau for M IGHV patients, suggesting that in these patients FCR may be curative. Last year, Shanafelt et al [69] published the results of their randomized phase III study (EGOC-ACRIN E1912 trial) comparing ibrutinib-rituximab versus FCR in young (<70 years) previously untreated CLL patients. Three-year PFS was not significant in patients with M IGHV patients (87.7% vs. 88% for the ibrutinib-rituximab and FCR groups respectively) however, was quite significant for those with UM IGHV (90.7% vs. 62.5%). Extended follow-up data (48 months) presented at the 2019 American Society of Hematology (ASH) Annual Meeting [71] favored IR over FCR with respect to PFS (hazard ratio [HR], 0.39; 95% CI, 0.23–0.57; *p* < 0.0001), and OS (HR, 0.34; 95% CI, 0.15–0.79; *p* = 0.009) in all patients. Subgroup analysis demonstrated improved PFS with IR in UM IGHV (HR 0.28; 95% CI, 0.17–0.48; *p* < 0.0001) and a trend towards improved PFS in M IGHV (HR, 0.42; 95% CI, 0.16–1.36; *p* = 0.086). We await the long-term results of this study to see if FCR still portends survival benefit in the age of novel targeted therapies for patients with M IGHV.

Alternative CIT regimens such as bendamustine plus rituximab (BR) and chlorambucil based therapy are commonly used in older, less fit CLL patients. In this regard, the three-arm multicenter phase III Alliance trial compared 547 older patients with previously untreated CLL to ibrutinib, ibrutinib plus rituximab or BR [72]. In subgroup analysis, patients with methylated ZAP70 (an established surrogate marker for M IGHV gene status) saw no improvement in PFS with single-agent ibrutinib compared to BR, arguing for acceptable use of BR in older patients with M IGHV CLL. Chlorambucil plus the anti-CD20 antibody obintuzumab has been shown in subgroup analyses of two large clinical trials to have similar efficacy to venetoclax plus obintuzumab and ibrutinib-based therapy in M IGHV patients and remains a reasonable option in older CLL patients [57,58].

Collectively, the results cited above make a strong argument for the role of IGHV gene mutational status as a predictive biomarker. We support the use of FCR in younger, fit patients with M IGHV given its potential for long-term remission and we recommend targeted therapy with a novel pathway inhibitor in patients with UM IGHV with case-by-case determinations being made in the setting of older and less fit CLL patients. Society guideline recommendations for the analysis of IGHV gene mutational status can be found in Table 2 [38,39,40,41].

### 3.3. NOTCH1 Gene Mutation

*NOTCH1* gene mutations occur in 10% of CLL patients at diagnosis and are enriched in subgroups carrying trisomy 12 or an unmutated IGHV gene status [20,23,24,26,73]. *NOTCH1* encodes for a transmembrane receptor which is constitutively expressed in CLL and most mutations affect the PEST domain of the NOTCH1 intracellular domain (NICD) resulting in its poor degradation and subsequent accumulation [74]. Upon receptor engagement, the NICD undergoes proteolytic cleavage and nuclear translocation forming an activator complex with the transcription factor RBPJ, leading to downstream activation of several pro-growth genes including *MYC* and *HES1* [75,76,77,78,79,80].

Patients with mutated *NOTCH1* do not appear to benefit from anti-CD20 targeted therapy. Results of the CLL8 trial showed that those 10% of patients harboring *NOTCH1* mutation responded the same after being treated with FC or FCR with respect to clinical response, MRD negativity, PFS or OS [44]. Patients without *NOTCH1* mutation, however, derived significant benefit from anti-CD20 targeted therapy with respect to the same endpoints.

To better elucidate the mechanism of anti-CD20 therapy refractoriness in the presence of mutated *NOTCH1*, our group investigated CD20 expression and relative lysis induced by anti-CD20 exposure in vitro in a series of 692 CLL in which 12% harbored mutated *NOTCH1* [81]. We observed significantly lower mean fluorescence intensity values of CD20 on flow cytometry in *NOTCH1* mutated cases compared to cases with wild-type *NOTCH1*. Furthermore, transcript levels of *MS4A1*, the gene encoding for CD20 [82] were lower in *NOTCH1* mutated than in *NOTCH1* wild type cases proportionate to mutational load. In vitro complement-dependent cytotoxicity assays demonstrated significantly lower % of relative lysis in *NOTCH1* mutated cases compared to *NOTCH1* wild type cases in the presence of rituximab and ofatumumab (*p* = 0.02 and 0.01, respectively).

Finally, using CLL-like cells transfected with the mutated *NOTCH1* intracellular domain (NICD-mut) we constructed a putative model for CD20 downregulation in *NOTCH1* mutated CLL whereby the truncated PEST domain of NICD-mut demonstrated increased affinity for RBPJ, thus tilting the balance between activation and repression complexes towards the former and allowing histone deactylase (HDAC) from the repression complex to freely migrate to other parts of the genome including the promotor of the *CD20* gene leading to downregulated transcription and CD20 expression [81].

Given this scenario, *NOTCH1* mutational status may be considered a promising predictive biomarker and when available, should be considered in all patients who are candidates for anti-CD20 targeted therapy. The cost and toxic effects of rituximab and obinutuzumab are considerable and should be avoided in *NOTCH1* mutated CLL particularly in the setting of CIT. Further clinical studies are needed to better define *NOTCH1* associated anti-CD20 chemorefractoriness in the setting of novel pathway inhibitors.

### 3.4. CD49d

CD49d is the α4 subunit of the integrin heterodimer α4β1 (VLA-4), a cell surface receptor that promotes microenvironment interactions of CLL leukemic cells [83,84,85]. VLA-4 has an important role in cell trafficking between blood and lymphoid organs as well as their survival and proliferation within the lymphoid organs by functioning as a cell-matrix and cell-cell receptor binding with the ligands VCAM-1, and fibronectin [86].

CD49d expression, defined by a cutoff of >30% is correlated with poorer survival in cohorts treated both with CIT and ibrutinib [19,87,88]. We recently published data demonstrating equally poor outcomes in the setting of CIT and ibrutinib in CLL patients with bimodal expression of CD49d—characterized by concomitant sub-populations of CD49d^pos^ and CD49d^neg^ clones—compared to homogenous CD49^neg^ CLL. Additionally, in cases of bimodal CD49d CLL treated with multiple lines of therapy, we observed an increase in the sub-population of CD49^pos^ cells, suggesting that CD49d expression plays a pivotal role in chemorefractoriness and disease potentiation. Homogenous CD49d^pos^/bimodal CD49d CLL showed reduced OS in all CLL-IPI risk categories except for the very high-risk group, arguing for its potential inclusion in updated prognostic calculators [88].

Given its role in microenvironment interactions, high CD49d expression might be expected to counteract the redistribution effect following administration of BCR pathway inhibitors of leukemic cells from the lymphoid compartment to the peripheral blood [56,89,90,91,92]. This observation is confirmed by a 2018 study by Tissino et al., demonstrating significantly decreased median % blood absolute lymphocyte count and lymph node mass reduction in CD49d positive cases after treatment with ibrutinib [87]. Furthermore, CD49d positive cases in this study showed independent negative prognostic capability with respect to PFS (HR [95% CI] 3.15 [1.16–8.53], *p* = 0.025) and CD49d expression further stratified PFS in the setting of IGHV mutational status and TP53 disruption. We also observed a shorter PFS in both CD49d^pos^ and bimodal CD49d cases compared to CD49d^neg^ cases in 158 patients treated with ibrutinib including 124 r/r cases (HR [95% CI] 2.63 [1.13–6.10], *p* = 0.024; 3.41 [1.32–8.79], *p* = 0.011 in r/r cases).

This data suggests a potent role of CD49d as a prognostic biomarker not only in the setting of CIT but also in the setting of novel BCR targeted therapy. In fact, given its capacity to inhibit canonical cell trafficking seen in the setting of BCR therapy, and its strong prognostic capacity in the setting of ibrutinib, CD49d appears a promising predictive biomarker. Ongoing studies are underway to further confirm the predictive role of CD49d expression.

### 3.5. DNA Methylation

Changes in the DNA methylation patterns are a molecular hallmark of tumorigenesis not only contributing to dysregulated gene expression; they are also strongly linked with cellular origin and memory of activity states [93]. In this regard, DNA methylation can identify the cellular origin in CLL clones and studies using whole-genome analysis have identified three distinct clinicobiologic subgroups: naïve B-cell-like (n-CLL), intermediate (i-CLL) and memory B-cell-like CLL (m-CLL) which differ not only in IGHV gene mutational status, but also with respect to clinical markers such as TTFT and OS [94,95]. UM IGHV is enriched in n-CLL (80–97%) and this group demonstrated poorer clinical courses compared to i-CLL and m-CLL particularly in early stage (Binet A/B) disease (TTFT: 3.1 years, 12.3 years and non-reached, respectively [*p* < 0.001]; 10 years OS: 52%, 96%, respectively [*p* < 0.001]) [95].

Recently, Wojdacz et al., applied these epigenetic classifications in the setting of CIT in a retrospective study of 605 treatment-naïve patients enrolled in three chemotherapy and CIT trials from the United Kingdom: (1) CLL4 which compared chlorambucil and fludarabine with or without cyclophosphamide [96], (2) ADMIRE which compared the efficacy of FCR against FCR and mitoxantrone [97], and (3) ARCTIC which compared FCR with FC mitoxantrone, and low-dose rituximab [98]. Multivariate Cox proportional analysis identified m-CLL as an independent prognostic factor for OS (HR, 0.46; 95% CI, 0.24–0.87; *p* = 0.018) in CLL4, and PFS (HR, 0.25; 95% CI, 0.10–0.57; *p* = 0.002) in ARCTIC and ADMIRE patients [99].

### 3.6. Complex Karyotype

In addition to the well-established recurrent cytogenetic aberrations with prognostic significance in CLL [100], complex karyotype (CK), defined by the presence of at least 3 numerical and/or structural abnormalities [101] is detectable in 14–35% of cases [102,103,104,105] and imparts further prognostic and potential predictive information both in the setting of CIT [49,103] and targeted therapy [60,106].

In a multicenter retrospective study, Baliakas et al., in conjunction with the European Research Initiative on CLL (ERIC), evaluated the impact of CK, identified by using chromosomal banding analysis (CBA), on OS in a cohort of 5479 treatment naïve CLL patients [102]. By dividing CK into high, intermediate and low groups with ≥5, 4 and 3 abnormalities, respectively (termed high-CK, intermediate CK; low-CK), and combining these with TP53 disruption and IGHV mutational status, they presented a novel hierarchical model bases on five distinct risk categories (from highest to lowest: high-CK; low-CK/intermediate-CK/TP53 disruption; non-CK/TP53 disruption; non-CK/nonTP53 disruption/UM IGHV; non-CK/nonTP53 disruption/M IGHV). Remarkably, they also found a low-risk cohort of patients with CK in combination with trisomy 12 and/or trisomy 19 with an exceptionally indolent profile giving rise to the notion that CK is not always a negative prognosticator.

Recently, the Italian group of Visentin et al., published data from a retrospective study of 522 CLL patients, the vast majority of whom received CIT, investigating the prognostic role of CK in combination with IGHV mutational status with respect to OS and TTFT [107]. CK was partitioned into two groups: those with major structural abnormalities (CK2) characterized by unbalanced translocations, chromosome addition, insertion, duplications, ring, dicentric and marker chromosome with a worse prognosis compared to other lesions (CK1) including balanced translocations, deletions, monosomies or trisomies. When combined with IGHV mutational status, 3 distinct risk categories emerged, from highest to lowest CK2, UM IGHV/CK1, and M IGHV/no CK with corresponding 5-year TTFT and OS of 31, 39 and 81% (*p* < 0.0001) and 67, 85 and 93% (*p* < 0.0001), respectively. Furthermore, median time to next treatment after FCR of BR was 1.86 and 4.79 years for CK2 and UM IGHV/CK1, but not reached for M IGHV/no CK patients (*p* < 0.0005), suggesting an emerging predictive role of CK in the CIT setting.

To discuss in details the functional mechanisms behind these prognosticators is behind the scope of the present review. However, some references addressing in details these aspects are the following [108,109,110,111,112,113,114,115].

## 4. Other Predictive Biomarkers in the Chemo-Free Era

### 4.1. BCR Pathway Mutations

BCR signaling is an important biologic feature of CLL tumor cells resulting in activity of the downstream regulators SYK, LYN, BTK and PI3K [116]. In this regard, BTK inhibitors (ibrutinib and acalabrutinib) which inhibit downstream intracellular signaling involving phospholipase Cγ2 (PLCg2), and PI3K inhibitors (idelalisib) which target a critical phosphorylation step in the signaling pathway, have revolutionized the treatment of CLL. Despite these gains however, refractoriness to BTK inhibition exists, largely owing to decreased responsiveness after a variable time on treatment.

Several point mutations in the BCR pathway have been identified in refractory cases. Point mutations such as *BTK* C481S disrupt covalent binding between ibrutinib and BTK and point mutations in PLCg2 disrupt downstream BCR signaling. These mutations are absent in ibrutinib naïve patients and appear to be selected for during BTK therapy [51,117,118,119].

### 4.2. BCL2 Mutations

BCL2 is a regulatory protein localized to the outer membrane of mitochondria that plays an important role in promoting cellular survival by binding pro-apoptotic proteins. BCL2 inhibitors act by binding BCL2, displacing pro-apoptotic proteins and promoting mitochondria-derived apoptosis through the release of molecules such as cytochrome C and reactive oxygen species [120]. BCL2 expression is elevated in 90% of patients with CLL [121] and the BCL2 inhibitor, venetoclax, has been an important addition to the compendium of novel treatments in previously treated CLL as monotherapy [53,63,122,123] or in combination with rituximab [124,125].

Despite high rates of clinical responsiveness, most patients who are heavily pretreated prior to starting venetoclax ultimately experience disease relapse or undergo Richter Transformation (RT) to diffuse large B-cell lymphoma [106,126]. Blombery et al., recently published data in a cohort of 67 patients with relapsed CLL treated with venetoclax [106] implicating the recurrent novel *BCL2* mutation, Gly101Val, in treatment refractoriness. The mutation, which reduces the affinity of venetoclax for BCL2 and confers acquired resistance in vitro and in vivo, was present in 7 of 15 paired samples at progression but not at treatment initiation [127]. Moreover, multiple novel *BCL2* mutations have been recently identified in parallel with *BCL2* Gly101Val during venetoclax therapy [128,129].

## 5. Conclusions

Herein we present a framework for the understanding of prognostic biomarkers and their predictive potential in the modern era of CLL. TP53 disruption, UM IGHV, *NOTCH1* mutation, and CD49d expression are the strongest prognosticators of disease progression and OS in CLL. Moreover, our recently published results on CD49d advocate for an updating of the CLL-IPI with the inclusion of this variable.

**Figure 1:**
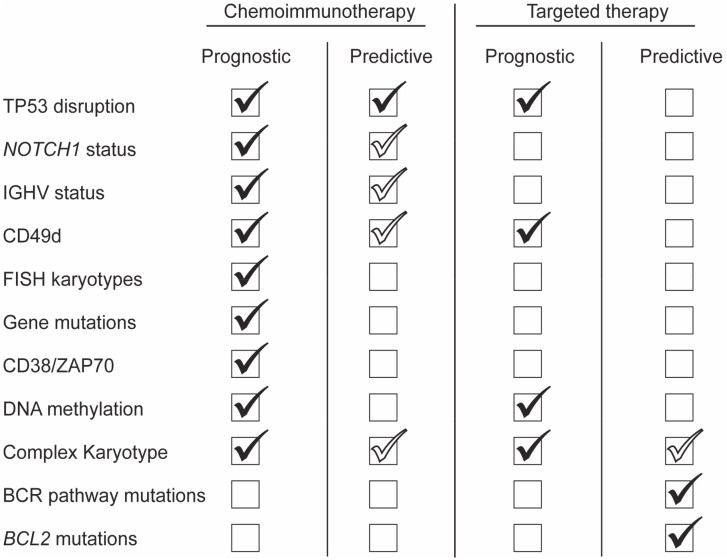
Prognosticators/Predictors in the context of chemoimmunotherapy and targeted therapy. Filled check marks identify biomarkers with a reported prognostic or predictive value in the setting of chemoimmunotherapy (left) or in the era of chemotherapy-free treatment with either Bruton’s tyrosine kinase, phosphoinositide 3-kinase inhibitors or B-cell lymphoma-2 (BCL2) inhibitors (right). In the context of chemoimmunotherapy, TP53 disruption is the only “true” predictive biomarker by consensus; other “proposed” predictive biomarkers are represented by empty check marks. TP53 disruption includes *TP53* mutation and/or del17p; FISH karyotype: del11q, trisomy12, normal cytogenetics and del13q; gene mutations: *BIRC3*, *SF3B1*. Abbreviations: IGHV, immunoglobulin heavy chain variable gene; ZAP70, zeta chain associated protein 70; BCR, B-cell receptor.

We recommend that CLL patients with TP53 disruption, including sub-clonal disease, and UM IGHV should not be treated with CIT and those with *NOTCH1* mutation should not be treated with anti-CD20 targeted therapy, particularly as part of a CIT regimen. The predictive significance of these markers should prompt clinicians to evaluate them in all CLL patients prior to treatment initiation and potentially at diagnosis to aid in both prognostication and potential early risk-adapted treatment in the setting of ongoing clinical trials (Figure 1).

CD49d and TP53 disruption also provide important prognostication in the setting of targeted therapy with BCR and BCL2 inhibitors and may in time develop into predictive biomarkers in this context as we learn more about the escape mechanisms driving progression (Figure 1). In the era of targeted therapy, we must continue to investigate new predictive biomarkers such as BCR and BCL-2 pathway mutations to identify those CLL patients with r/r disease who should be considered for treatment in new clinical trials.

## Figures and Tables

**Table 1:** Today’s most well-established prognostic biomarkers in chronic lymphocytic leukemia.

Category	Biomarkers
Host Factors	Age, Gender, Ethnicity
Disease Markers	Lymph node involvement (size, site(s) of involvement), Hepatomegaly, Splenomegaly, LDT, WBC count, ALC, Anemia, Thrombocytopenia,
Antigen Expression	CD38, ZAP70, CD49d/VLA-4
Serology	β2M, TK, LDH, IL-8
Genetics	del17p, *TP53* mutation, del11q, del13q, trisomy 12, *NOTCH1* mutation, DNA methylation, complex karyotype, *SF3B1* mutation, *BIRC3* mutation, *BRAF* mutation, miR-223, miR-29c, miR-155
Immunogenetics	IGHV sequence, BCR structure

Abbreviations: LDT, lymphocyte doubling time; WBC, white blood cell; ALC, absolute lymphocyte count; ZAP70, zeta chain associated protein 70; VLA-4, vascular leukocyte adhesion molecule-4; β2M, Beta-2 microglobulin; TK, thymidine kinase; LDH, lactate dehydrogenase; IL-8, interleukin 8; miR, microRNA; IGHV, immunoglobulin heavy chain variable gene; BCR, B-cell receptor.

**Table 2:** Guideline recommendations for TP53 and *IGHV* analysis in clinical practice.

Society	Recommendation	Timing
iwCLL		
TP53 disruption	Always	Prior to treatment
IGHV gene mutational status	Always	Prior to treatment
BCSH		
TP53 disruption	Always	Prior to treatment
IGHV gene mutational status	“Should be considered”	Prior to treatment
NCCN		
TP53 disruption	Always	At diagnosis or prior to treatment ^1^
IGHV gene mutational status	Always	At diagnosis or prior to treatment
ESMO		
TP53 disruption	Always	Prior to treatment
IGHV gene mutational status	“Desirable”	Prior to treatment

TP53 disruption includes both del17p by fluorescent in-situ hybridization and *TP53* gene mutational analysis by either Sanger or next-generation sequencing. ^1^ In the case of analysis performed in early-stage disease under a “watch-and-wait” strategy or relapsed/refractory cases undergoing subsequent therapy, TP53 analysis should be repeated prior to treatment to assess effects of clonal evolution. Abbreviations: *IGHV*, immunoglobulin heavy chain variable gene; iwCLL, international workshop on chronic lymphocytic leukemia; BCSH, British committee for standards in haematology; NCCN, national comprehensive cancer network; ESMO, European society for medical oncology.
